# Frequency of cervical premalignant lesions in the gynecologic patients of a tertiary hospital in Mogadishu, Somalia

**DOI:** 10.1186/s12905-022-02106-0

**Published:** 2022-12-07

**Authors:** Sabri Kurtay, Khadija Yusuf Ali, Ahmed Issak Hussein

**Affiliations:** Somali Mogadişu Türkiye Recep Tayyip Erdoğan Training and Research Hospital, Mogadishu, Banadır Somalia

**Keywords:** Cervical cancer, Human Papilloma Virus (HPV), Somalia, Sub-Saharan Africa, Women’s health

## Abstract

**Background:**

Cervical cancer is the fourth most common cancer worldwide and is the most frequently diagnosed cancer in 23 countries and the most common cause of death in 36 countries, mostly from Sub-Saharan African countries. Cervical screening is a key element to reduce the incidence and mortality of cervical cancer. Cancer screening is low in Sub-Saharan Africa. This study aims to provide information about cervical premalignant lesions frequency in Somalia.

**Methods:**

The pathology results of cervicovaginal smear samples obtained from patients aged 25–65 years who applied to the gynecology outpatient clinic between October 5 and December 5, 2021 were analyzed retrospectively. SPSS 22.0 was used for the statistical analysis of the data.

**Result:**

Among the 497 results, 63 premalignant lesions were detected. The rate of premalignant lesions (63/497) was found to be 12.3%. The most common premalignant lesion was atypical squamous cells of undetermined significance (ASC-US).

**Conclusion:**

In this study, the frequency of cervical premalignant lesions in Somalia was found to be higher than in the literature. Vaccination, screening, and early diagnosis are the most important components in the fight against cervical cancer. Access to vaccination, screening, and early diagnosis, which are the most important components in the fight against cervical cancer in Somalia, will be possible with the cooperation of the national health system and international organizations.

## Background

Cervical cancer is the fourth most common cancer worldwide, the most frequently diagnosed cancer in 23 countries, and the most common cause of death in 36 countries, mostly in Sub-Saharan African countries [1]. According to World Health Organisation (WHO), cervical cancer cases are expected to increase until 2030. More than 85% of cases are among young, low-education women living in the poorest countries in the world. 604,000 women were diagnosed with cervical cancer, and more than half of them died from the disease in 2020 globally [2]. WHO's Global Strategy includes eliminating cervical cancer by the end of the century, and this is the first worldwide cancer-related initiative, which includes screening and vaccination [3]. If these targets are achieved in 2030, the reduction rate in the incidence of cervical cancer is expected to be 2%, 42% and 97% in 2030, 2045 and 2120, respectively, and as a result, 74 million cases are expected to be prevented [2].

US Preventive Services Task Force (USPSTF) recommends screening women with cytology or hr-HPV (high-risk) tests against cervical cancer [4]. The American Cancer Society (ACS) recommends Human Papilloma Virus (HPV) testing every 5 years between the ages of 25–65. In cases where HPV testing is not possible, ACS recommends screening with HPV co-testing every 5 years or cytology every 3 years between the ages of 25–65 [5]. American College of Obstetricians and Gynecologists (ACOG) recommends HPV testing or testing with acetic acid in low-income countries where cytology is not available [6].

Although cervical cancer screening is crucial in reducing mortality and morbidity [7], Sub-Saharan Africa screening rates are low in Sub-Saharan Africa. [7] The highest death of cervical cancer was noted in Somalia, Kiribati, Eritrea, and the Central African Republic between 2007 and 2017 [8]. There is no national strategy for cervical cancer screening in Somalia. The rates of benefiting from cervical cancer screening programs are low in Ethiopia and Kenya (5.5% and 16.4%, respectively) [9]. Somalia, on the other hand, does not have data to compare with the data of these neighboring countries. This study was planned to investigate the frequency of cervical premalignant lesions in Somalia.

## Material and method

The study was carried out in Somalia Mogadishu Turkey Recep Tayyip Erdogan Training and Research Hospital (MRTE-TREH) between October 5, 2021 and December 5, 2021. Ethical clearance and permission were obtained from the Institutional Review Board. Written and signed informed consent was obtained from all participants and/or their legal surrogates. Cervical smear results of patients who applied to the gynecology outpatient clinic within 3 months were retrospectively analyzed. Gynecological patients aged 25–65 years were included in the study. Those with menstrual bleeding during the examination and pregnant women were excluded from the study. Cervical smear specimens were collected using an endocervical brush, spread on a slide, and fixed with an alcohol-based solution. Cervical smear results of the patients were classified according to the Bethesda 2014 system [10]. The frequency of premalignant lesions was evaluated. Categorical variables were compared with the binomial test. A value of *p* < 0.05 was considered significant. The analyses were performed using IBM SPSS Statistics Version 22.0.

## Results

Cervical smear results of 497 patients who met the criteria within 3 months were evaluated. Among these results, 63 premalignant lesions were detected. 50 atypical squamous cells of undetermined significance (ASC-US), 6 atypical squamous cells cannot exclude HSIL (ASC-H), 3 atypical glandular cells (AGC), 2 low-grade squamous intraepithelial lesion (LSIL) and 2 high-grade squamous intraepithelial lesion (HSIL) were detected. The results of 434 patients were negative for intraepithelial lesion or malignancy (NILM). The rate of premalignant lesions (63/497) was found to be 12.3%. (Table 1) The most common premalignant lesion was ASC-US. (Table 2).

**Table 1:** Frequency of cervical premalignant lesions in Somalia

	Count	%
Premalignant	63	12.3
Negative for lesion or malignancy	451	87.7
Total	497	100

**Table 2:** Distribution of premalignant results

	Count	%
ASC-US	50	79.4
ASC-H	6	9.5
AGC	3	4.8
LSIL	2	3.2
HSIL	2	3.2
Total	63	100.0

## Discussion

The prevalence of premalignant lesions worldwide varies depending on countries' cervical cancer screening strategies. [11, 12] In a study examining the data of approximately 1 million women, it is seen that the frequency of premalignant lesions is 4.4% [13]. Our study revealed that the frequency of premalignant lesions was 12.3% in Somalia. This rate is statistically significantly higher than the literatüre (*p* < 0.05). In Somalia, where the effects of the civil war still continue, there is no national screening program for cervical cancer. Pathological examination is available in the hospital where the study was conducted. Thus, information was obtained about the frequency of cervical premalignant lesions from cervical smears of patients who may be related to cervical pathology.

The lack of data about cervical cancer in low- and middle-income countries is one of the most important barriers to cancer screening. [14] The reason why cervical cancer is more common in low- and middle-income countries than in high-income countries is the lack of screening programs in these countries [15]. The present study reveals the frequency of cervical premalignant lesions.

The cost-effectiveness of screening, which is effective in the prevention of cervical cancer, was examined among countries with different income levels, and it was seen that HPV DNA was the most effective screening method in almost all countries examined [16]. HPV testing has shown to be effective in low-income countries, as well [17]. In a review examining the effectiveness of the self-sampling screening method, it was stated that this method was generally effective in cervical cancer screening [18]. Self-testing of HPV is an effective and applicable method, especially in high-income countries, and increases the success of screening [19, 20]. Based on this information, cervical cancer screening with the HPV DNA method can be considered a reasonable option for Somalia in terms of feasibility and cost-effectiveness.

The sensitivity of the visual inspection with acetic acid (VIA) method in detecting cervical intraepithelial lesion-2 (CIN-2) lesions is 16.6–82.6%, the specificity is 82.1–96.8%, and this method provides both diagnosis and treatment at the same visit. [21] In another study, although it was stated that both HPV testing and VIA methods are effective in screening in Sub-Saharan Africa, it was claimed that the main effective struggle should be based on population-based screening and vaccination. [22] Comparing the screening methods available in Sub-Saharan Africa, VILI (Visual inspection with Lugol’s iodine) was found to be more sensitive than VIA and more affordable and simple than cytology [23]. VILI/VIA screening, which is applied in low-income countries, is also a cervical cancer screening method that can be applied in Somalia.

In low-income countries, screening alone is not enough. Standardized laboratories and specialized equipment and personnel are required to carry out the cervical cancer screening program at the national level [24]. Considering the low number of obstetricians in Somalia, the role of physicians and midwives working in primary care will be high in screening. The level of knowledge of the target population and the perception of cancer are also important factors. WHO recommended incorporating the HPV vaccine into national vaccination programs in 2009, and over 100 countries have done so by 2020 [25]. Therefore, a screening and vaccination program that can take into account cultural characteristics in Somalia and be integrated into the local health system will be effective in combating cervical cancer. The factors affecting cervical cancer screening of immigrants, including Somalis, in Finland were examined. Although the rate of participation in cervical cancer screening is at the lowest level in Somali women, the most important factor affecting participation in screening is expressed as having had a gynecological examination within the last 1 year. [26] Including a screening method to the gynecological examination in Somalia will enable early diagnosis of cervical cancer.

### Limitation

Included patients were selected from symptomatic women. The availability of data from asymptomatic women will provide more information about the frequency of premalignant lesions. Another limitation of the study is that the relationship between patient characteristics and premalignant lesions was not clearly evaluated. Prospective studies are needed to examine this relationship.

## Conclusion

In this study, it was observed that the frequency of premalignant cervical lesions in Somalia is higher than in many countries. The absence of a vaccination and screening program against cervical cancer in the country is the main reason for this high frequency. The establishment of national screening and prevention strategies is imperative to combat cervical cancer. On the other hand, in line with the goals of the World Health Organization to eliminate cervical cancer, international programs and financial arrangements will contribute to reducing the incidence of cervical cancer in Somalia.

## Data Availability

The datasets generated and/or analyzed during the current study are available from the corresponding author upon reasonable request.

## Abbreviations

ACOG: American College of Obstetricians and Gynecologists
ACS: American Cancer Society
AGC: Atypical glandular cells
ASC-H: Atypical squamous cells cannot exclude HSIL
ASC-US: Atypical squamous cells of undetermined significance
CIN: Cervical intraepithelial lesion
HPV: Human Papilloma Virus
HSIL: High-grade squamous intraepithelial lesion
LSIL: Low-grade squamous intraepithelial lesion
MRTE-TREH: Somalia Mogadishu Turkey Recep Tayyip Erdogan Training and Research Hospital
NILM: Negative for intraepithelial lesion or malignancy
USPSTF: United States Preventive Services Task Force on cervical cancer screening
WHO: World Health Organisation
VIA: Visual inspection with acetic
VILI: Visual inspection with Lugol’s iodine
